# Retrospective study on prevention of bladder neck contracture by local injection of betamethasone after transurethral resection of the prostate in patients with small-volume prostate

**DOI:** 10.3389/fsurg.2025.1726670

**Published:** 2026-01-21

**Authors:** Qiang Wang, Yunlong Jiang, Ping Ao, Houbao Huang, Wenqiang Zhang, Xiaoxu Yuan

**Affiliations:** 1 Department of Urology, The First Affiliated Hospital of Wannan Medical College (Yijishan Hospital of Wannan Medical College), Wuhu, Anhui, China; 2 Department of Urology, Zhuhai People's Hospital (Zhuhai Hospital Affiliated with Jinan University), Zhuhai, Guangdong, China

**Keywords:** betamethasone, bladder neck contracture, small-volume prostate, surgical complications, transurethral resection of prostate

## Abstract

**Objective:**

Bladder neck contracture (BNC) is a challenging postoperative complication of transurethral resection of the prostate (TURP), especially in patients with small-volume prostates (<40 mL) who are at high risk. This retrospective study aimed to evaluate the efficacy and safety of local betamethasone injection in preventing BNC following TURP in this specific population.

**Methods:**

Clinical data of 248 patients with small-volume benign prostatic hyperplasia (BPH) who underwent TURP at Zhuhai People's Hospital from January 2017 to December 2023 were retrospectively analyzed. Patients were divided into two groups: the betamethasone injection group (*n* = 128) receiving 8 mg betamethasone injected into the submucosal layer of the bladder neck (3, 6, 9, and 12 o'clock positions) during surgery, and the control group (*n* = 120) undergoing TURP without betamethasone injection. All procedures were performed using standardized bipolar plasma TURP without bladder neck incision. Baseline characteristics, intraoperative parameters, and postoperative outcomes were collected. The primary endpoint was the incidence of BNC within 12 months of follow-up, diagnosed based on clinical symptoms, uroflowmetry (maximal urine flow <10 ml/sec), and cystoscopy. Secondary endpoints included the incidence of other postoperative complications.

**Results:**

The baseline characteristics of the two groups were comparable (all *p* > 0.05). During the 12-month follow-up, the incidence of BNC in the betamethasone injection group was significantly lower than that in the control group (2.3% vs. 10.8%, *p* = 0.004). Multivariate logistic regression analysis identified local betamethasone injection as an independent protective factor against BNC (OR = 0.20, 95% CI: 0.06-0.69, *p* = 0.011), while prostate volume ≤30 mL was an independent risk factor (OR = 3.21, 95% CI: 1.08-9.53, *p* = 0.036). There were no significant differences in the incidence of other postoperative complications (urinary tract infection, secondary hemorrhage, urethral stricture, urinary incontinence) between the two groups (all *p* > 0.05).Conclusion: Local injection of betamethasone during TURP significantly reduces the incidence of BNC in patients with small-volume prostates without increasing perioperative complications. This intervention targets the inflammatory and fibrotic mechanisms underlying BNC and serves as a safe and effective adjuvant strategy to optimize surgical outcomes in this high-risk population.

## Introduction

Benign prostatic hyperplasia (BPH) is a prevalent benign condition in middle-aged and elderly men, characterized by progressive enlargement of the prostate gland (1). It often leads to bladder outlet obstruction (BOO) and lower urinary tract symptoms (LUTS), significantly impairing patients’ quality of life (2–4). Transurethral resection of the prostate (TURP) remains a cornerstone of surgical intervention for BPH, especially in patients with small-volume prostates, where it effectively relieves obstructive symptoms (2, 5–7). However, postoperative complications—among which bladder neck contracture (BNC) (8, 9)is a notable and challenging one—continue to affect clinical outcomes.

BNC is defined as the abnormal narrowing of the bladder neck following transurethral procedures, with reported incidence ranging from 0% to 9.6% in different cohorts (8, 10, 11). It typically presents with progressive dysuria, decreased urinary flow rate, and even recurrent urinary retention, often necessitating reoperation and thus increasing the physical and economic burden on patients. Accumulating evidence has identified several risk factors for BNC, among which smaller preoperative prostate volume stands out as a well-recognized independent predictor (8, 12–14). Patients with small-volume prostates are particularly susceptible to BNC after TURP, possibly due to the higher risk of excessive resection or iatrogenic injury to the bladder neck during surgery, which triggers local inflammatory responses and subsequent scar formation (15, 16).

Currently, strategies for preventing BNC mainly focus on optimizing surgical techniques, such as preserving the bladder neck mucosa and avoiding excessive electrocautery (17, 18). However, these measures do not fully eliminate the risk, especially in high-risk populations like those with small-volume prostates (19, 20). Betamethasone, a potent glucocorticoid, exhibits strong anti-inflammatory properties and can inhibit fibroblast proliferation and collagen synthesis (21), thereby reducing scar tissue formation at the surgical site (22). Local injection of betamethasone at the bladder neck during or after TURP may potentially mitigate the inflammatory cascade and prevent the development of BN (20),, yet relevant clinical evidence in patients with small-volume prostates remains limited.

In this retrospective non-randomized study, local betamethasone injection during TURP significantly reduced BNC in patients with small-volume prostates without increasing perioperative complications.

## Patients and methods

### Inclusion and exclusion criteria

Clinical data of patients with benign prostatic hyperplasia (BPH) who underwent transurethral resection of the prostate (TURP) at Zhuhai People's Hospital from January 2017 to December 2023 were retrospectively collected.

Inclusion criteria were: (1) Preoperative diagnosis of BPH confirmed by clinical symptoms, digital rectal examination, and transrectal ultrasound; (2) Preoperative prostate volume < 40 ml (measured by transrectal ultrasound using the formula: volume = length × width × height × 0.52); (3) First-time TURP; (4) Complete clinical and follow-up data; (5) Patients were divided into two groups based on whether they received local betamethasone injection during surgery: the betamethasone injection group and the control group (without betamethasone injection).

Exclusion criteria were: (1) Severe preoperative urinary tract infection, primary urethral or bladder neck stenosis, or neurogenic bladder; (2) History of prostate or urethral surgery; (3) Comorbid prostate cancer; (4) Incomplete clinical data.

### Surgical procedures

**TURP technique:** All procedures were performed by urologists with more than 5 years of experience in transurethral surgery. Under spinal or general anesthesia, patients were placed in the lithotomy position. All TURP procedures adopted bipolar plasma energy and were conducted using the Olympus Prostate Plasma Resection System (Olympus Corporation, Tokyo, Japan), which includes the Olympus URF-V2 resectoscope and ESG-400 electrosurgical generator. The working mode was set to saline-based bipolar plasma, with consistent operational parameters: 320 W for pure resection, 200 W for coagulation, and a high frequency of 430 KHZ ± 20%. This modality was selected for its superior hemostatic effect and reduced risk of transurethral resection syndrome (TURS) compared to monopolar energy, as supported by prior clinical evidence.
The resectoscope working element used was the Olympus standard loop electrode (model: A22161A). Its energy return pathway transmits through the resectoscope sheath (model: A22150A; 26Fr outer diameter, low-friction coating) rather than the loop itself, minimizing local thermal damage to the bladder neck via optimized current dispersion. The loop electrode features a 5 mm working length and 12 mm width, enhancing surgical precision while reducing mechanical trauma to the bladder neck. A resectoscope was inserted transurethrally to visualize the prostate, bladder neck, and ureteral orifices. Hyperplastic prostate tissue was resected using the aforementioned bipolar electrode, with meticulous attention to avoid excessive resection of the bladder neck to minimize iatrogenic injury. Hemostasis was achieved via electrocautery during and after resection.
**Local betamethasone injection:** In the betamethasone injection group, after completing TURP and confirming adequate hemostasis, local injection of betamethasone was performed. A 22-gauge injection needle was inserted through the working channel of the resectoscope. Betamethasone (4 mg/ml) was injected into the submucosal layer at the 3, 6, 9, and 12 o'clock positions of the bladder neck, with 0.5 ml injected at each site (total dose 8 mg). The injection was conducted under direct visualization to ensure precise delivery. The control group underwent TURP using the same technique but without betamethasone injection.

### Clinical data collection and BNC diagnostic criteria

The following data were extracted from electronic medical records:

Preoperative data: age, body mass index (BMI), smoking history, comorbidities (hypertension, diabetes mellitus, etc.), prostate-specific antigen (PSA) level, prostate volume (measured by transrectal ultrasound within 1 week before surgery), urine leucocyte count, urine culture results, serum creatinine (SCr), estimated glomerular filtration rate (eGFR), and preoperative catheterization status;
Intraoperative data: surgical duration, resected prostate volume;
Postoperative data: duration of catheterization, occurrence of bladder neck contracture (BNC), and other complications.

The diagnostic criteria for BNC were consistent with previous reports: BNC was suspected in patients with progressive dysuria, and confirmed by cystoscopy when urodynamics showed a decreased maximal urine flow (<10 ml/sec). Patients were categorized into BNC and non-BNC groups based on the development of BNC after surgery.

### Follow-up

All patients were followed up for at least 12 months postoperatively. Follow-up assessments included inquiry about lower urinary tract symptoms, uroflowmetry, and cystoscopy if necessary to detect BNC.

### Statistical analysis

SPSS 23.0 software was used for data analysis. Normally distributed data were presented as mean ± standard deviation (SD), and non-normally distributed data as median (1st quartile, 3rd quartile). Comparisons between the betamethasone injection group and the control group were performed using Student's t-test (for normally distributed data), Mann–Whitney *U* test (for non-normally distributed data), or chi-square test (for categorical data) as appropriate. Multivariate logistic regression analysis was used to identify independent factors associated with BNC. Statistical significance was defined as *p* < 0.05.

## Results

A total of 248 patients with small-volume prostate (prostate volume <40 ml) who underwent TURP at Zhuhai People's Hospital from January 2017 to December 2023 were included. Among them, 128 patients were in the betamethasone injection group (local injection of 8 mg betamethasone during surgery), and 120 patients were in the control group (without betamethasone injection).

The baseline characteristics of the two groups were comparable (Table 1). In the betamethasone group, the mean age was 62.3 ± 5.6 years, with a median prostate volume of 29.1 (23.8, 35.4) ml; the control group had a mean age of 61.9 ± 6.0 years, with a median prostate volume of 28.9 (23.5, 36.1) ml. There were no statistically significant differences in age, prostate volume, preoperative PSA, smoking history, comorbidities (hypertension, diabetes mellitus), positive preoperative urine culture, or other variables between the two groups (all *p* > 0.05).

**Table 1:** Baseline characteristics of patients in the betamethasone injection group and control group.

Parameter	Betamethasone injection group (*n* = 128)	Control group (*n* = 120)	*P* value
Age (years), mean ± SD	62.3 ± 5.6	61.9 ± 6.0	0.572
Preoperative prostate volume (ml), median (IQR)	29.1 (23.8, 35.4)	28.9 (23.5, 36.1)	0.813
Preoperative PSA (ng/ml), median (IQR)	3.7 (2.0, 6.4)	3.9 (2.2, 6.7)	0.654
BMI (kg/m^2^), mean ± SD	24.0 ± 2.2	23.8 ± 2.4	0.526
Smoking history, *n* (%)	47 (36.7)	43 (35.8)	0.886
Hypertension, *n* (%)	40 (31.3)	37 (30.8)	0.927
Diabetes mellitus, *n* (%)	14 (10.9)	13 (10.8)	0.982
Positive preoperative urine culture, *n* (%)	24 (18.8)	23 (19.2)	0.931
Preoperative urine leucocyte (3+), *n* (%)	18 (14.1)	17 (14.2)	0.985
Postoperative catheterization days, median (IQR)	9 (7, 11)	9 (6, 11)	0.598
Surgical time (min), median (IQR)	35 (32, 38)	36 (31, 39)	0.789

Data are presented as mean ± standard deviation (SD) for normally distributed variables, median (interquartile range, IQR) for non-normally distributed variables, or counts (percentages) for categorical variables. Comparisons were performed using Student's *t*-test, Mann–Whitney *U* test, or chi-square test as appropriate. IQR: 1st quartile, 3rd quartile; PSA: prostate-specific antigen; BMI: body mass index.

During the 12-month follow-up, the overall incidence of BNC in the entire cohort was 6.5% (16/248). Notably, the incidence of BNC in the betamethasone injection group was significantly lower than that in the control group (2.3% vs. 10.8%, *p* = 0.004). Specifically, 3 cases of BNC were identified in the betamethasone group (3/128), while 13 cases occurred in the control group (13/120).

No severe complications related to betamethasone injection were observed in the betamethasone group. The most common postoperative complication in both groups was urinary tract infection, with an incidence of 10.9% (14/128) in the injection group and 12.5% (15/120) in the control group, showing no statistically significant difference (*p* = 0.687). Other complications such as secondary hemorrhage, urethral stricture, and urinary incontinence were rare and comparable between the two groups (Table 2).

**Table 2:** Postoperative complications in the betamethasone injection group and control group.

Complication	Betamethasone injection group (*n* = 128), *n* (%)	Control group (*n* = 120), *n* (%)	*P* value
Bladder neck contracture (BNC)	3 (2.3)	13 (10.8)	0.004
Urinary tract infection	14 (10.9)	15 (12.5)	0.687
Secondary hemorrhage	5 (3.9)	4 (3.3)	0.801
Urethral stricture	2 (1.6)	3 (2.5)	0.652
Urinary incontinence	7 (5.5)	6 (5.0)	0.863
Any complication	31 (24.2)	33 (27.5)	0.564

Data are presented as counts (percentages). Comparisons were performed using the chi-square test.

Multivariate logistic regression analysis revealed that local injection of betamethasone was an independent protective factor against BNC (OR = 0.20, 95% CI: 0.06–0.69, *p* = 0.011), while small-volume prostate (≤30 ml) was confirmed as an independent risk factor for BNC (OR = 3.21, 95% CI: 1.08–9.53, *p* = 0.036).

The results of the present study demonstrated that local injection of betamethasone during TURP significantly reduced the incidence of BNC in patients with small-volume prostate, with no increase in other postoperative complications.

## Discussion

Bladder neck contracture (BNC) remains a challenging complication after transurethral endoscopic surgery for benign prostatic hyperplasia (BPH) (8, 9), particularly in patients with small-volume prostates, as smaller preoperative prostate volume has been identified as an independent risk factor for BNC (12, 13, 23, 24). The present retrospective study evaluated the efficacy of local betamethasone injection in preventing BNC after TURP in patients with small-volume prostates (<40 ml), and the results demonstrated that the 1-year incidence of BNC was significantly lower in the betamethasone injection group (2.3%) compared with the control group (10.8%), while other postoperative complications were comparable between the two groups. These findings provide clinical evidence for the role of local glucocorticoid injection in reducing BNC risk in high-risk populations.

The observed significant reduction in BNC incidence with local betamethasone injection is attributed to glucocorticoids’ targeted effects on BNC's multi-stage fibroproliferative pathophysiology. Previous studies have linked BNC pathogenesis to local inflammation and excessive scarring (25, 26), triggered by factors like TURP-induced bladder neck trauma, preoperative urinary tract infection, or prolonged catheterization (12). Specifically, BNC progresses through three core stages (27): 1. Inflammatory phase: TURP-related ggmechanical/thermal injury recruits neutrophils, macrophages, and lymphocytes, which release pro-inflammatory mediators (e.g., prostaglandins) and pro-fibrotic cytokines (e.g., TGF-β1, PDGF) to initiate fibrosis; 2. Fibroblast activation phase: Pro-fibrotic cytokines drive fibroblast proliferation and differentiation into myofibroblasts, leading to excessive type I/III collagen synthesis and disorganized scar formation; 3. Contracture phase: α-SMA-expressing myofibroblasts generate contractile forces, while collagen cross-linking and elastic fiber loss reduce tissue compliance, causing bladder neck narrowing. As a potent glucocorticoid, betamethasone interrupts this cascade at multiple key links. It modulates the inflammatory microenvironment by inhibiting immune cell infiltration, downregulating pro-inflammatory/pro-fibrotic factors (e.g., interleukin-6, tumor necrosis factor-α) (21, 28–30), and stabilizing lysosomal membranes to prevent further tissue damage. For fibrosis, it suppresses fibroblast activation, myofibroblast differentiation, and collagen synthesis (22, 31), while boosting matrix metalloproteinase (MMP-1, MMP-3) activity to degrade excess extracellular matrix. Additionally, it inhibits pathological angiogenesis via downregulating VEGF and bFGF, reducing vascular density and nutrient supply for scar growth. By targeting these processes, betamethasone limits hypertrophic scarring at the bladder neck—BNC's core pathological feature. This mechanism aligns with prior findings that inflammation-related factors (e.g., positive preoperative urine culture) drive BNC (32), further supporting local betamethasone intervention in high-risk patients.

Notably, the overall BNC incidence in our cohort (2.3% in the injection group and 10.8% in the control group) falls within the range reported in previous studies (0%–9.6%), with the control group rate reflecting the higher risk in small-volume prostate populations and the injection group demonstrating a clinically meaningful reduction. Previous studies have also highlighted that surgical techniques (e.g., avoiding excessive resection of the bladder neck) (33) are critical for reducing BNC risk (34); in our study, all TURP procedures were performed by experienced surgeons with standardized techniques to minimize iatrogenic injury, which likely contributed to the baseline risk profile. The additional benefit of betamethasone injection further supports its role as an adjuvant measure in high-risk patients (e.g., those with small-volume prostates) (13).

Regarding safety, the comparable incidence of other postoperative complications (e.g., urinary tract infection, secondary hemorrhage, urethral stricture) between the two groups indicates that local betamethasone injection does not increase adverse events. This is clinically relevant, as previous studies have noted that complications such as bleeding and urinary tract infection are common after transurethral procedures (35), and our results suggest that adding betamethasone injection does not exacerbate these risks. The absence of significant differences in complication profiles supports the feasibility of integrating this intervention into routine TURP practice for small-volume prostates.

Several limitations of the present study should be acknowledged. First, as a retrospective study, it is susceptible to selection bias, despite efforts to balance baseline characteristics between groups. Second, long-term follow-up beyond 1 year is needed to confirm the durability of the protective effect. Third, the optimal dosage and injection timing of betamethasone were not explored, which warrants further investigation in prospective randomized controlled trials.

Transurethral bladder neck incision (TUBNI) was not performed in any of the patients in this study. Although TUBNI may hold potential for BNC prevention, it is inherently an invasive procedure that carries inherent risks such as urethral injury, bleeding, and other complications. In the absence of conclusive evidence of benefit, we did not routinely perform this procedure in enrolled patients.

In conclusion, local injection of betamethasone during TURP significantly reduces the incidence of BNC in patients with small-volume prostates, without increasing other postoperative complications. This intervention targets the inflammatory and fibrotic mechanisms underlying BNC, complementing the surgical techniques emphasized in previous studies. These findings provide preliminary support for local betamethasone injection as a potential strategy to improve outcomes in high-risk patients with small-volume BPH, with further validation needed for its safety and effectiveness.

## Data Availability

The original contributions presented in the study are included in the article/Supplementary Material, further inquiries can be directed to the corresponding authors.
